# Sciellin mediates mesenchymal-to-epithelial transition in colorectal cancer hepatic metastasis

**DOI:** 10.18632/oncotarget.8264

**Published:** 2016-03-22

**Authors:** Chuan-Kai Chou, Chi-Chen Fan, Pei-Shan Lin, Pei-Yu Liao, Jia-Chen Tung, Chang-Hsun Hsieh, Mien-Chie Hung, Chung-Hsuan Chen, Wei-Chao Chang

**Affiliations:** ^1^ National Applied Research Laboratories, National Laboratory Animal Center, Taipei, Taiwan; ^2^ Superintendent Office, Mackay Memorial Hospital, Taipei, Taiwan; ^3^ Department of Medical Laboratory Science and Biotechnology, Yuanpei University, Hsinchu, Taiwan; ^4^ Center for Molecular Medicine, China Medical University Hospital, Taichung, Taiwan; ^5^ Graduate Institute of Cancer Biology, China Medical University, Taichung, Taiwan; ^6^ Department of Orthopaedic Surgery, National Taiwan University Hospital, National Taiwan University, Taipei, Taiwan; ^7^ Department of Molecular and Cellular Oncology, The University of Texas MD, Anderson Cancer Center, Houston, TX, USA; ^8^ Genomics Research Center, Academia Sinica, Taipei, Taiwan; ^9^ Institute of Biochemistry & Molecular Biology, National Yang-Ming University, Taipei, Taiwan; ^10^ Department of Chemistry, National Taiwan University, Taipei, Taiwan; ^11^ Institute of Atomic & Molecular Sciences, Academia Sinica, Taipei, Taiwan

**Keywords:** colorectal cancer, hepatic metastasis, mesenchymal-to-epithelial transition, SCEL

## Abstract

Hepatic metastasis is the major cause of mortality in colorectal cancer (CRC) patients. Using proteomic analysis, we found sciellin (SCEL) to be specifically expressed in hepatic metastatic CRC cell lines. SCEL knockdown increased CRC cell migration and invasion, while overexpression had the opposite effect. SCEL knockdown also caused cancer cells to form more invasive structures within 3D cultures, increased the mesenchymal marker vimentin, and attenuated the epithelial marker E-cadherin. SCEL increased WNT signaling by activating β-catenin and its downstream target c-myc, and activated mesenchymal-to-epithelial transition (MET) through a SCEL-β-catenin-E-cadherin axis. SCEL showed higher expression in late stage primary CRC than in its hepatic metastatic counterpart. SCEL expression is dynamically modulated by TGF-β1 and hypoxia, revealing a plastic MET mechanism for tumor colonization. Intrahepatic injection in immunodeficient mice revealed that SCEL is necessary for metastatic CRC tumor growth in the liver. These results demonstrate that SCEL is a MET inducer dynamically regulated through the metastasis process. They suggest SCEL may be a useful therapeutic target for preventing or eliminating CRC hepatic metastasis.

## INTRODUCTION

Colorectal cancer (CRC) is the second leading cause of cancer death in the developed world [1]. Approximately 50% of patients die within 5 years of diagnosis due to recurrent disease and metastasis [2]. After the lymph nodes, the liver is the most frequent site of CRC metastasis. The median survival of patients with untreated but potentially resectable metastases is 8 months and the 5-year survival rate of these patients is less than 5% [3].

For metastatic invasion to occur, cancer cells lose their epithelial characteristics and acquire the behavior of mesenchymal cells, a process known as epithelial-to-mesenchymal transition (EMT), which enables them to migrate and invade other tissues [4]. EMT is controlled by three families of transcription factors: Snail (Snail/Slug), ZEB (ZEB1/ZEB2), and Twist1 [5]. During EMT, cancer cells switch off the expression of epithelial markers such as E-cadherin and turn on the expression of mesenchymal markers such as vimentin [6]. EMT is usually linked with stemness properties, including apoptosis resistance, transient quiescence, and self-renewal capacities, revealing cancer stem cell involvement in metastasis [7, 8]. For clonal outgrowth at metastatic sites, the reverse process mesenchymal-to-epithelial transition (MET) is thought to be required for proliferation and differentiation of cancer cells [9]. MET was originally proposed based on the observation that carcinoma metastases usually present a well-differentiated epithelial phenotype [10]. The concept of transient EMT-MET switches in metastasis was not proved until recently [11], and the need for a MET for efficient metastasis is supported by experimental data [12, 13]. Compared to EMT, however, very few MET inducers have been identified [14–16].

Sciellin (SCEL), a precursor of the cornified envelope [17], contains 16 inexact repeats of 20 amino acids and a LIM domain (derived from LIN-11, Isl1, and MEC-3) that may function as a protein interaction module regulating the localization of SCEL and protein assembly in the cornified envelope [18]. SCEL is an arterial intima-enriched protein that contributes to the stress properties of stratified epithelium [19, 20]. In this study, we demonstrated that SCEL is necessary for CRC hepatic colonization and that EMT stimulators, such as TGF-β and hypoxia, decreased SCEL expression, suggesting plasticity in EMT-MET switches.

## RESULTS

### SCEL is highly expressed in hepatic metastasis CRC cell lines

To identify novel target proteins involved in CRC hepatic metastasis, membrane proteins from four CRC cell lines, SW480, SW620, L1, and L2, were extracted and analyzed using mass spectrometry. CRC cell lines SW480 and SW620 were derived from the same patient; SW480 was isolated from the primary tumor and SW620 from a lymph node metastasis [21]. Cell lines L1 and L2, derived from SW620, displayed liver-specific metastasis in a xenograft model [22]. Two biological replicates were used and two technical replicates for each sample were performed. In total, 2099 proteins were identified, including 1772 membrane proteins. Among the membrane proteins, 316 were plasma membrane proteins based on Gene Ontology definition. The proteins highly expressed in L1 or L2, but not detected in SW480 are listed in Table 1.

**Table 1:** proteins overexpressed in hepatic metastasis CRC cell lines L1 and L2

			Proteomic analysis (ratio)					
Uniprot	Protein Name	Gene Name	MW[kDa]	SW480	SW620	L1	L2	PEP^(a)^
P52799	Ephrin-B2 (LERK5)	EFNB2	36.92	0^(b)^	1^(c)^	3.15	5.49	< 1.00E-307
O43490	Prominin-1 [CD133]	PROM1	97.20	0	1	5.85	2.83	5.68E-304
Q99541	Perilipin-2 (ADFP)	PLIN2	48.08	0	1	5.32	2.46	1.09E-143
P19440	Gamma-glutamyltranspeptidase 1 [CD224]	GGT1	61.41	0	1	4.17	17.70	3.89E-29
O95171	Sciellin	SCEL	77.55	0	0	∞^(d)^	∞	3.54E-127
Q8NFJ5	Retinoic acid-induced protein 3 (RAIG1)	GPCR5A	40.25	0	0	∞	∞	2.67E-66
P50281	Matrix metalloproteinase-14	MMP14	65.89	0	0	∞	∞	3.48E-16
P08582	Melanotransferrin [CD228]	MFI2	80.21	0	0	0	∞	6.53E-75

The identified proteins highly expressed in both L1 and L2, but not detected in SW480 are included in this list.

P.S.

(a) Posterior error probability (PEP) was obtained from statistical analysis of total peptide identification for a protein in one sample. The value essentially operates as a statistical value, and low PEP indicates high statistical significance.

(b) The value 0 represents that the mass intensity of indicated protein is below detection limitation.

(c) The mass intensity of indicated protein in SW620 served as control and was set as 1. The expression ratio of L1 and L2 is relative to SW620.

(d) The symbol ∞ represents that the indicated protein is detectable in L1 or L2, but its mass intensity is below detection limitation in SW620.

**Figure 1:**
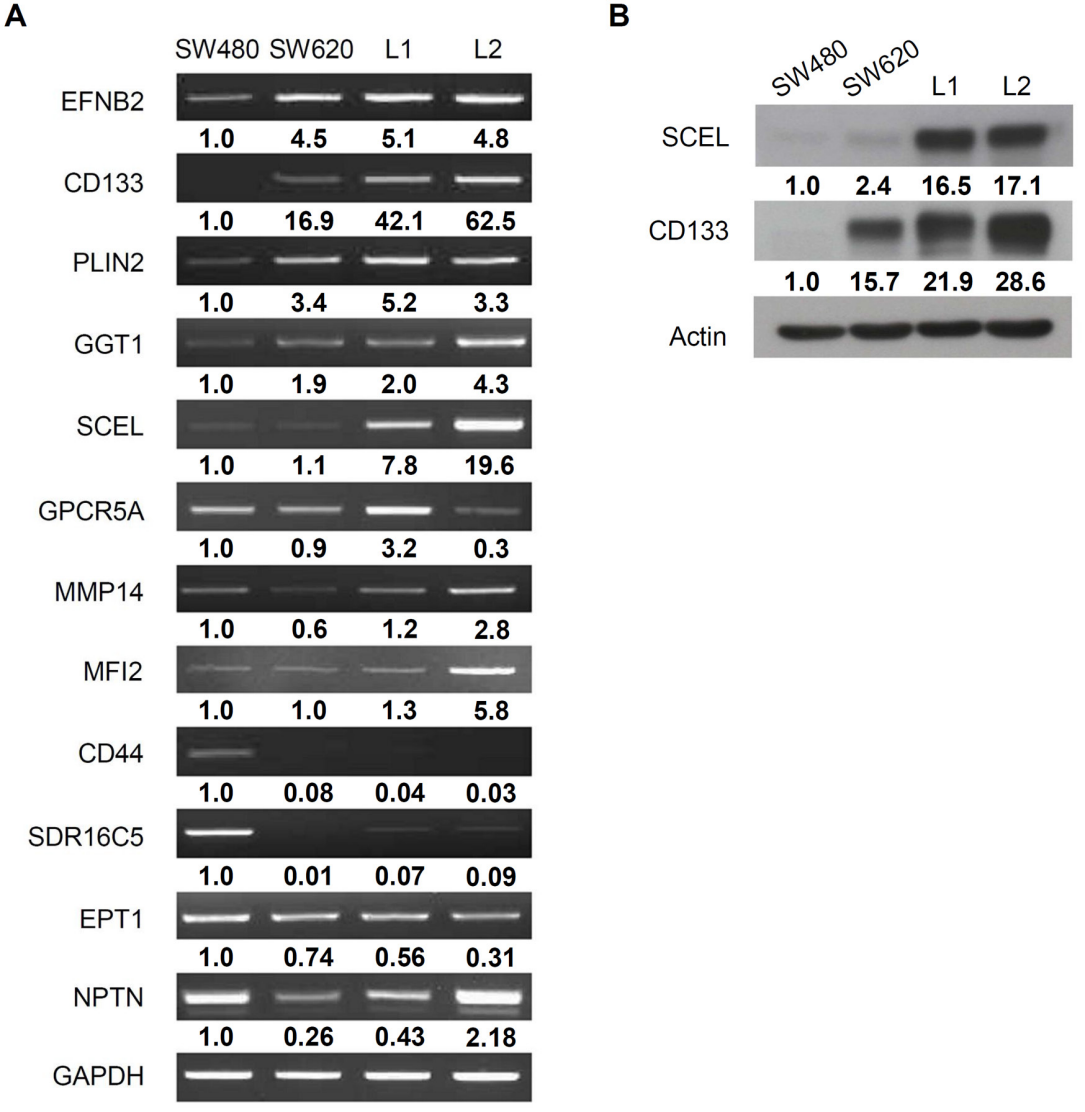
Validation of proteomic result **A.** RT-PCR assay for the proteins overexpressed in hepatic metastatic CRC cell lines L1 and L2. GAPDH served as control. **B.** Western blot assay for SCEL and CD133. β-actin served as loading control. The quantification was normalized to control GAPDH or β-actin, and the value of SW620 was set as 1.

The mRNA levels of CD133 and SCEL, measured by RT-PCR, agreed with the proteomic finding (Figure 1A). Western blot assays of CD133 and SCEL were also consistent with the proteomic results (Figure 1B). CD133 is a well known cancer stem cell-related marker in numerous types of cancers including colorectal cancer [23, 24] and an efficient prognostic marker with higher expression predicting poorer clinical outcome [25]. Moreover, CD133 plays vital roles in cancer cell growth, differentiation, metabolism, and metastasis [26]. CD133 was highly expressed in metastatic CRC cell lines SW620, L1, and L2, indicating that CD133 is a common metastatic factor. Notably, SCEL protein levels were higher in hepatic metastatic cancer cells L1 and L2, implying that SCEL specifically contributes to CRC hepatic metastasis.

### SCEL mediates MET properties in CRC metastasis

**Figure 2:**
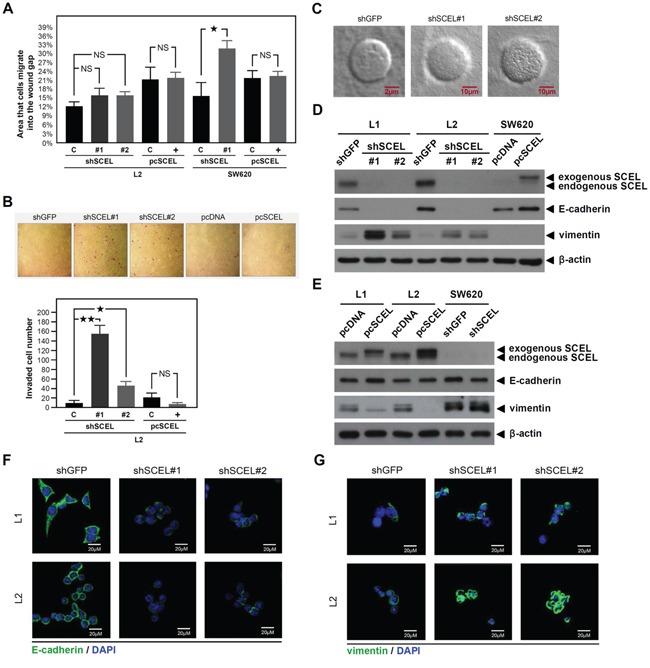
SCEL increases MET properties of CRC Two kinds of shRNAs were used for SCEL knockdown experiment, shRNA#1 target sequence was: GCACAAGGAAATCAAGATGAA and shRNA#2 target sequence was: CATTGAAGATCAACTCTTGTA. SCEL overexpression was performed using pcDNA3.1/myc-His vector. **A.** Wound-healing assay. CRC cells were cultured in the ibidi Culture-Insert well, and the migration images of cancer cells were recorded at 0 (removal of Culture-Insert) and 16 hr. Migration distance was measured using ImageJ. Experiments were performed in triplicate (mean ± SD). **B.** Matrigel invasion assay. Representative photographs show the crystal violet-stained invading cancer cells of L2 (controls, SCEL knockdown or overexpression). Experiments were performed in triplicate, and the numbers of invading cells were quantified (mean ± SD). **C.** Collagen gel 3D culture assay. Tumor sphere formation was induced by suspending cancer cells in agarose coated culture dish with serum free medium for 7 days. Tumor spheres were then harvested and resuspended in collagen I solution with growth medium. After 14 days, the 3D structures of cancer cells were observed using confocal microscopy. Representative photographs show the 3D structures of the control and SCEL knockdown of L2 cells. **D.** and **E.** The effects of SCEL knockdown or overexpression on EMT marker expression in L1, L2, and SW620 were determined using western blot. β-actin served as loading control. **F.** The expression of epithelial marker E-cadherin (green) and **G.** The expression of mesenchymal marker vimentin (green) in the control (shGFP) and SCEL knockdown (shRNA#1 and shRNA#2) of L1 and L2 were analyzed using confocal microscopy. DAPI (blue) was used for nuclear stain.

SCEL is one component of cornified envelope, a rigid structure that provides physical resistance and acts as a water barrier [27], which suggests that SCEL increases the rigidity, or cell stiffness, of colorectal cancer cells. Recent studies demonstrate that metastatic cancer cells from cell lines and patients are more pliable than non-metastatic cells [28–30]. The related information raises a controversial question: if SCEL increases cancer cell stiffness and reduces metastatic potential, why is SCEL so highly expressed in hepatic metastasis CRC cell lines L1 and L2? We speculated that SCEL could serve as a MET inducer, promoting CRC hepatic colonization. We used short hairpin RNAs (shRNAs) to knock down SCEL expression and exogenously expressed SCEL in SW620, L1, and L2; we evaluated the effects on cancer cell migration and invasion, the *in vitro* metastatic properties. SCEL knockdown increased CRC cell migration, whereas overexpression had the opposite effect. Only the changes resulting from SCEL knockdown in SW620 reached statistical significance (Figure 2A). SCEL knockdown increased whereas overexpression reduced L2 cell invasiveness (Figure 2B). 3D collagen gel culture assay revealed that SCEL knockdown caused cancer cells to form more invasive structures compared to the compact sphere of control cells (Figure 2C). These results suggest that expression SCEL reduces the metastatic abilities of CRC cells. To further examine SCEL as a potential MET inducer, we investigated the effect of SCEL on the expression of the epithelial marker E-cadherin and the mesenchymal marker vimentin in CRC cells. SCEL knockdown reduced E-cadherin and increased vimentin levels in Sw620, L1, and L2 (Figure 2D, 2E). Confocal microscopy assays of the expression of E-cadherin and vimentin in L1 and L2 (Figure 2F, 2G) were consistent with western blot results (Figure 2D). Taken together, our observations strongly suggest that SCEL is a potential MET inducer for CRC hepatic metastasis.

### SCEL activates Wnt signaling pathway

**Figure 3:**
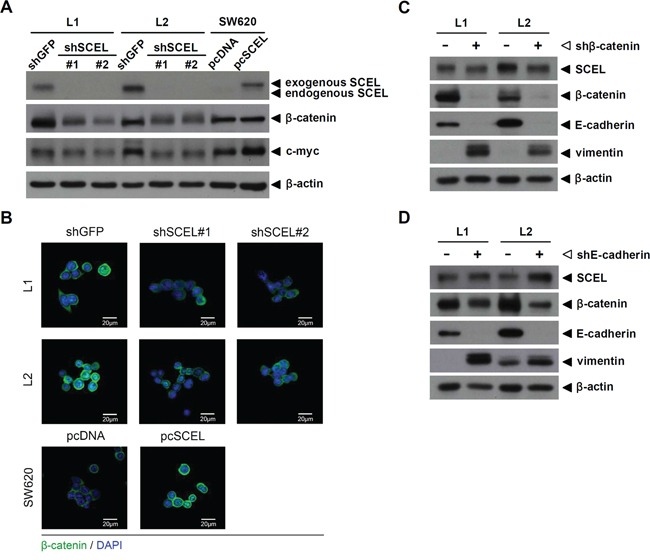
SCEL activates the Wnt signaling pathway **A.** The protein expression of β-catenin and its target gene c-myc was analyzed using western blot assay. β-actin served as loading control. **B.** The expression of β-catenin (green) in L1, L2, and SW620 was analyzed using confocal microscopy. DAPI (blue) was used for nuclear stain. **C.** and **D.** Western blot to determine the effect of β-catenin or E-cadherin knockdown on EMT marker expression in L1 and L2. β-actin served as loading control.

The Wnt cascade controls intestinal epithelium homeostasis, and CRC has been considered a disease of defective Wnt signaling [31]. Activation of the Wnt/β-catenin signaling pathway plays a pivotal role in initiating CRC development and in cancer growth and metastasis [32, 33]. A recent study demonstrated that over 94% of colorectal cancers had a mutation in the Wnt/β-catenin signaling pathway [34], resulting in the accumulation of β-catenin and increased activity of c-myc [35]. Due to the potential role of SCEL in cancer metastasis, we wondered whether SCEL was involved in regulation of the Wnt signaling pathway. We examined the expression of β-catenin and its target gene product c-myc using western blot assay. Protein levels of β-catenin and c-myc were reduced in L1 and L2 along with SCEL knockdown, and SCEL overexpression increased the protein expression of β-catenin and c-myc in SW620 (Figure 3A). The expression of β-catenin in L1, L2, and SW620 was confirmed by confocal microscopy (Figure 3B). To further investigate the potential signaling pathway in SCEL-mediated MET, we separately knocked down β-catenin and E-cadherin expression in L1 and L2 cells. β-catenin knockdown did not affect SCEL levels, but reduced E-cadherin and increased vimentin levels (Figure 3C). E-cadherin knockdown also did not affect SCEL levels, but partly reduced β-catenin and increased vimentin levels (Figure 3D). Collectively, these data suggested that SCEL activates the MET process through the SCEL-β-catenin-E-cadherin axis.

### SCEL expression correlates with malignancy

**Figure 4:**
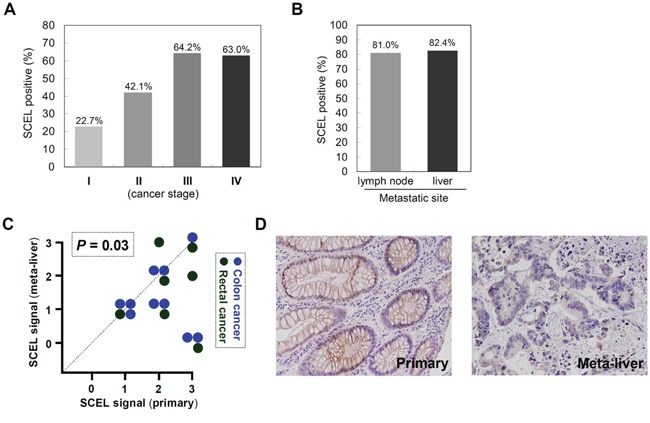
SCEL differentially expressed in primary colorectal cancer and metastatic liver **A.** The expression of SCEL in various stages of CRC was examined using tissue array CO1501 (US Biomax, Maryland, USA). A pathologist in CMUH was consulted for the interpretation of IHC results. The IHC signals were scored as 0, 1, 2, and 3; a score ≧1+ indicated positive detection. **B.** SCEL expression in CRC specimens with lymph node metastasis (*N*=21) or with hepatic metastasis (*N*=17) was determined using IHC analysis. **C.** The paired tumor specimens (primary and metastatic) from the same patient were analyzed using IHC (*N*=17). *P* value was calculated by Wilcoxon signed ranks test. **D.** Representative photographs showed the IHC staining of SCEL in primary colorectal cancer and its hepatic metastatic counterpart.

To evaluate SCEL expression in clinical samples, we performed immunohistochemical staining (IHC) with anti-SCEL antibody on the colorectal cancer tissue array CO1501 (US Biomax). IHC illustrates that the percentage of cell expressing SCEL were 22.7%, 42.1%, 64.2%, and 63% in cancer stage I, II, III, and IV of CRC, respectively (Figure 4A), revealing SCEL expression correlates with cancer malignancy. IHC on clinical specimens from CMUH indicated that SCEL was expressed in 81.0% (17/21 cases) of CRC specimens with lymph node metastasis and in 82.4% (14/17 cases) of CRC specimens with hepatic metastasis (Figure 4B), consistent with the previous finding that SCEL expression correlated with cancer malignancy. We therefore performed IHC to examine SCEL expression using paired tissues of primary CRC and its corresponding liver metastasis. Surprisingly, all primary CRC tumors showed SCEL expression, and most had similar or higher SCEL levels than their hepatic counterparts (*P*=0.03) (Figure 4C, 4D). Our analysis implies that SCEL expression in primary colorectal cancer is necessary for hepatic metastasis.

### TGF-β1 and hypoxia inhibit SCEL expression

**Figure 5:**
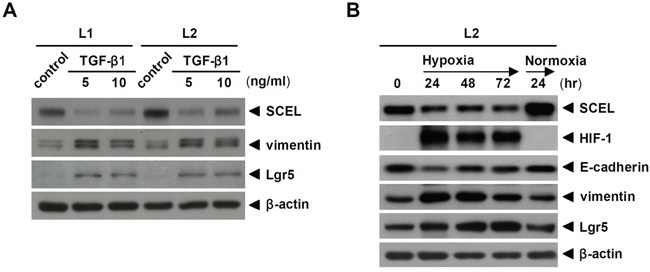
TGF-β1 and hypoxia inhibit SCEL expression **A.** L1 and L2 were treated with 5 and 10 ng/mL TGF-β1 individually for 24hr. The expression of SCEL, vimentin, and Lgr5 was determined using western blot. **B.** L2 was cultured in hypoxic condition for 3 days and then restored to normoxic condition for 1 day. The expression of SCEL, HIF-1, E-cadherin, vimentin, and Lgr5 was determined using western blot. β-actin served as loading control.

Since SCEL is originally expressed in primary colorectal cancer tissue, we wondered whether SCEL expression was dynamically regulated during metastasis. Oxygen deficiency is a common feature of tumor microenvironment, especially at later stages of tumor progress. Hypoxic tumor microenvironment enhances cancer stem cell properties [36] and promotes the metastatic phenotype [37]. Transforming growth factor-beta (TGF-β) is a pluripotent cytokine with dual tumor-suppressive and tumor-promoting effects. In later stages of colorectal cancer, TGF-β promotes cancer cell migration, invasion, angiogenesis, and metastasis [38, 39]. To explore the regulation of SCEL during cancer metastasis, we treated cancer cells with TGF-β1 or cultured cancer cells under hypoxic condition to promote metastatic characteristics. TGF-β1 treatment increased the expression of the mesenchymal marker vimentin and the cancer stem cell marker Lgr5 [40], and reduced SCEL levels in both L1 and L2 (Figure 5A). Hypoxia gradually reduced SCEL expression in cancer cells, while the levels of vimentin and Lgr5 were increased and the epithelial marker E-cadherin was reduced. When cancer cells were transferred back to normoxia, higher expression of SCEL and E-cadherin and lower expression of vimentin and Lgr5 were restored (Figure 5B). These results indicate plasticity of SCEL function during metastasis, in which metastatic cancer cells (or cancer stem cells) achieve a more mesenchymal phenotype by reducing SCEL expression.

### SCEL promotes CRC hepatic colonization in xenograft mouse model

**Figure 6:**
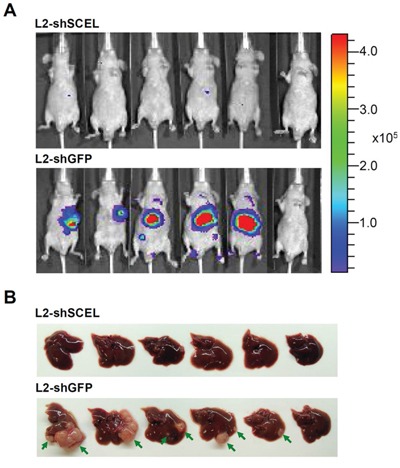
SCEL promotes CRC hepatic colonization in xenograft mouse model **A.** L2 cells (1×10^5^ cells/50 μL) were inoculated into BALB/c nude mice (BALB/cAnN.Cg-*Foxn1^nu^*/CrlNarl) using intrahepatic injection. The growth of tumor was monitored by bioluminescence imaging using IVIS imaging system. Representative IVIS images of mice at 8^th^ week after injection. **B.** Mice were sacrificed at 8^th^ week after injection, and the morphology of livers are presented. Tumor masses are indicated by green arrow.

**Figure 7:**
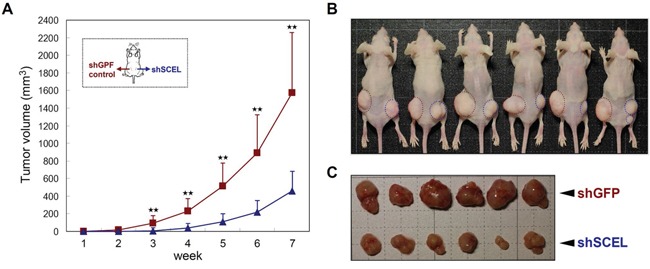
SCEL knockdown attenuates CRC tumorigenesis in subcutaneous tumor model The control (shGFP) and SCEL knockdown (shSCEL) L2 cells (1×10^6^) were injected subcutaneously into both flanks of 5-week-old male BALB/c nude mice. **A.** Tumor sizes were recorded weekly and the average tumor volume of each group was plotted. Error bars show standard derivation. The observation was continued for eight weeks after inoculation. **B.** Tumors in each mouse were labeled with dashed circles; red represents shGFP and blue represents shSCEL. **C.** Morphologies of tumors formed in each group.

The previous study has demonstrated that both L1 and L2 cell lines have increased ability to form hepatic metastasis compared with the parental SW620 cells [22]. To characterize the functions of SCEL on CRC hepatic colonization, we used control (shGFP) and SCEL knockdown L2 cells to perform intrahepatic injection in BALB/c nude mouse system. SCEL knockdown reduced the hepatic colonization ability of L2 cells (Figure 6A, 6B). Although SCEL knockdown increases metastatic ability of L2 cells, shSCEL L2 cells growing in other organs were not observed in this experiment, suggesting that SCEL knockdown does not change the organ tropism of CRC cells. To further exclude the effect of SCEL on increasing cell growth conferring CRC hepatic colonization, we performed *in vitro* cell proliferation assay. SCEL knockdown did not significantly change *in vitro* cell proliferation of L1 and L2 cells (Supplementary Figure 1). Additionally, we subcutaneously injected control (shGFP) and SCEL knockdown L2 cells into both flanks of immunodeficient mice and monitored tumor growth. SCEL knockdown reduced the speed of tumor growth, but did not completely stop the growth of L2 cells (Figure 7A, 7B, 7C). Taken together, these results demonstrate that SCEL is necessary for CRC hepatic colonization.

## DISCUSSION

Metastasis is a complex process involving multistep regulation of gene expression [41]. EMT-MET switch is the most popular theory to explain the common observation that metastatic nodules histologically resemble the epithelial phenotype of primary tumor [42]. Unlike EMT, current studies provide too little information to understand the essence of MET, including when MET inducer expression turns on if needed, how cancer cells resist the mechanical force to invade and colonize into the ordered metastasized tissue, and why cancer cells select to settle in a given organ (organotropic metastasis), etc. In previous studies, researchers usually induced MET by silencing EMT-related transcription factors or proteins [12, 13]; whether any MET inducer was involved in this process remains elusive. In this study, we identified a novel MET inducer SCEL from liver-specific metastasis CRC cell lines L1 and L2 [22]. SCEL has several features of a MET inducer, including promoting epithelial phenotype and increasing epithelial molecular marker expression as well as reducing cancer cell migration and invasion abilities. SCEL knockdown deprives CRC cells of hepatic colonization ability in xenograft mouse model.

Given that SCEL is one of the cornified envelope proteins, we propose its potential rigidity could increase cell stiffness and provide physical resistance at the metastatic site. A variety of techniques such as atomic force microscopy and microfluidic devices show that metastatic cancer cells are less stiff than non-metastatic cells [28]. Due to the clinical correlation between cell stiffness and cancer progression and metastasis, tumor stiffness is viewed as a prognostic biomarker for metastasis prediction [43]. Additionally, variant hardness of environmental materials could produce different interactions with cancer cells to affect their phenotype and behavior. For example, soft extracellular matrix and tissue-like material prevent stable cell-to-cell adherens junction formation, promote MMP activity, and induce invasion phenotype [44–46]. Therefore, in addition to being a mechanical property, higher cell stiffness could change the interactions of cancer cells with cells of the metastasized tissue and contribute to the epithelial characteristics of cancer cells for metastatic colonization. Further studies are in progress to determine the effects of SCEL expression on cell stiffness and matrix interaction.

The identification of SCEL-associated proteins may provide some clues to its functions in cancer cells. We performed MS to identify SCEL-associated proteins (Supplementary Table 1). 21 total proteins were identified most with phosphorylation (18 proteins) or acetylation (15 proteins) modifications based on database search in DAVID Bioinformatics Resources (https://david.ncifcrf.gov/).

Previous studies indicated that nuclear localization of β-catenin and canonical Wnt signaling were increased at the invasive front of CRC [47], but the most dramatic increase in nuclear β-catenin was during MET [15]. In this study, we found that SCEL knockdown reduced the expression of β-catenin as well as its nuclear target gene c-myc, showing that SCEL, as a MET inducer, is indeed able to regulate the Wnt signaling pathway and β-catenin expression. The decrease in β-catenin is not restored by LiCl or chloroquine treatment (data not shown), revealing that SCEL-induced elevation of β-catenin is not through GSK3β or autophagy.

The MET process has been assumed necessary for later steps in metastasis. It is surprising to find MET inducer SCEL expressed in primary tumor from clinical specimens, and that it is necessary for hepatic metastasis. However, it is not a unique observation because another well characterized MET inducer miR-200s is also detected in primary tumor [11]. This observation leads to a hypothesis that the full spectrum of MET is controlled by a dynamic plasticity of MET inducers. In the current study, we demonstrated that SCEL is dynamically regulated under TGF-β1 treatment and hypoxic condition, which are known to promote the mesenchymal phenotype and cancer cell invasiveness. Moreover, changing the culture condition back to normoxia restores the elevated SCEL expression level. This dynamic mechanism lets cancer cells compromise the intrinsic functions of a MET inducer for primary tumor escape and metastatic colonization.

In our study design, SW480 and SW620 were used to represent primary adenocarcinoma and lymph node metastasis colorectal cancer cells, respectively. We determined the expression of SCEL in clinical specimens to further confirm its disease relevance and found SCEL in the early stages of colorectal cancers (Figure 4A). In addition, SCEL showed a higher ratio of expression in the later stages of colorectal cancer, suggesting SCEL expression correlates with cancer progression. Since SCEL acts as a MET inducer, it is expected to be found in higher levels at the metastatic site relative to primary tumor, like the expression of the key MET mediator E-cadherin in breast cancer and prostate [48, 49]. Surprisingly, we observed that primary colorectal cancer cells express similar or higher SCEL levels than its hepatic counterpart in clinic specimens, implying the MET process involves more complex control beyond our intuitive speculation. Thinking about plasticity and different expression of SCEL in clinical specimens, our study raises several important questions. Do circulating cancer cells express lower levels of SCEL? Why are some cancer cells unable to restore SCEL to the original levels of primary tumor at the metastatic site? Do the relatively lower levels of SCEL represent further metastatic potential from colonized site?

Collectively, we discovered a novel MET inducer SCEL responsible for colorectal cancer hepatic metastasis. SCEL's dynamic expression throughout metastasis hints at plastic regulation involving the ENT-MET switches. Although it acts as a MET inducer, SCEL is highly expressed in primary colorectal cancer before metastasis onset. According to integrated proteomics database, SCEL is only detected, with low expression, in a few tissues including cervix and esophagus. The characteristics of SCEL make it a suitable target candidate for low side effect targeted therapies to prevent or eliminate colorectal cancer metastasis.

## MATERIALS AND METHODS

### Cell lines and cell culture

Colorectal cancer cell lines SW480 and SW620 were isolated from the tissue of a Caucasian male. SW480 was derived from primary adenocarcinoma, and SW620 was derived from lymph node metastasis one year later [21]. Cancer cell lines L1 and L2 were isolated from hepatic metastasis of SW620 in a mouse xenograft model [22]. SW480 was cultured in L15 medium (Gibco) and SW620, L1, and L2 were cultured in DMEM/F12 medium (Gibco); both L15 and DMEM/F12 media were supplemented with 10% (v/v) fetal bovine serum (Gibco), 100 U/mL penicillin, and 100 mg/mL streptomycin (Gibco). For hypoxic culture, cancer cells were cultured in a CO_2_ incubator maintained at 94% N_2_, 5% CO_2_, and 1% O_2_.

### Membrane protein extraction and tryptic peptide preparation

Membrane proteins were extracted from cancer cells using compartmental protein extraction kits CNM (BioChain Institute). First, cytoplasmic and nucleic proteins were extracted and removed by reaction buffers containing HEPES, MgCl_2_, KCl, sucrose, glycerol, and sodium orthovanadate. Next, membrane proteins were extracted by NP40 and sodium deoxycholate. Membrane proteins were subjected to in-gel enzymatic digestion into peptides prior to mass spectrometry analysis. For in-gel digestion, membrane proteins were separated by SDS-PAGE and divided into ten gel fractions, which were then cut into small gel pieces (<1mm^3^) individually. The in-gel digestion procedure includes coomassie blue destaining, disulfide-bond reduction, acrylation with iodoacetamide, and trypsin digestion, following our previously described method [50].

### Mass spectrometry analysis

Membrane proteins were identified and quantified using the linear ion trap-Fourier transform ion cyclotron resonance mass spectrometer (LTQ-FTICR MS, Thermo Fisher). The survey scan of MS analysis (*m/z* 320-2,000) was performed on LTQ-FTICR MS with a mass resolution of 100,000 at *m/z* 400. Top ten most abundant multiply charged ions were sequentially isolated for MS/MS by LTQ. MaxQuant [51] and MaxLFQ [52] software were used for protein identification and label-free quantification by normalization and maximal peptide ratio extraction methods. The significance threshold for the identification was set to *P* < .01.

### Wound healing and matrigel invasion assay

For *in vitro* migration assay, cancer cells (70μL; concentration: 7×10^5^ cells/mL) were added to Culture-Insert well (ibidi) and cultured for 24 hr. After removal of Culture-Insert, cancer cells were cultured for 20 hr. The migration distance of cancer cells was recorded and measured using ImageJ.

For *in vitro* invasion assay, cancer cells (1.5×10^5^ cells in 200 μL) were suspended in DMEM medium and added to the upper half of a PET membrane transwell insert chamber (BD Biosciences), which was coated with Matrigel (1 mg/mL; BD Biosciences) on a 24-well plate. DMEM medium supplemented with 10% FBS was added as a chemoattractant to the lower half. After incubation at 37°C for 24 hr, cancer cells that passed through the insert were fixed with 3.7% formalin (Sigma-Aldrich) and stained with 0.1% crystal violet (Sigma-Aldrich).

### Western blot analysis

Proteins were separated using 10% SDS-PAGE, and then transferred onto a nitrocellulose membrane using electroblot at 400V at 4°C for 3 hr in 25 mmol/L Tris-HCl, 197 mmol/L glycine, and 13.3% (v/v) methanol. Membranes were blocked with 5% (w/v) skim milk in TBST for 1 hr, and incubated with primary antibodies at room temperature overnight. After gently agitating in three TBST washes and one change TBS wash for 15 min each, horseradish peroxidase-conjugated secondary antibodies were further incubated at room temperature for 1 hr. Immunoreactive signals were revealed using an enhanced ECL substrate according to the manufacturer's instructions (NEN Life Science). The primary antibodies used in this study included SCEL (H00008796-B01P; Abnova), CD133 (PAB12663; Abnova), E-cadherin (#5296; Cell Signaling), vimentin (#3932; Cell Signaling), β-catenin (#9562; Cell Signaling), c-myc (710007; Invitrogen), HIF-1 (610958; BD Biosciences), Lgr5 (GTX62071; GeneTex), and β-actin (ab8226; abcam).

### Immunohistochemistry analysis

Tissue specimens were obtained from the Department of Pathology of China Medical University Hospital (CHUH) in compliance with protocols approved by the CMUH institutional review board (CMUH102-REC1-116). Paired samples from primary colorectal tumor and its hepatic metastasis counterpart were used (17 pairs, *N*=34). The expression of SCEL in clinical specimens was examined using immunohistochemical (IHC) staining. Briefly, mouse anti-human SCEL antibody (B01P; Abnova) was used to perform IHC staining using horseradish peroxidase-conjugated avidin-biotin complex from the Vectastain Elite ABC Kit (Vector Laboratories) and AEC chromogen (Vector Laboratories). The sections were counterstained with hematoxylin and mounted. All staining results were evaluated by experienced histologists.

### Intrahepatic inoculation and subcutaneous inoculation of mouse model

The animal procedure (103-21-N) was approved by the Institutional Animal Care and Use Committee (IACUC) at CHUH. For intrahepatic inoculation, CRC control group (shGFP) and SCEL knockdown experimental group (shSCEL) were mixed with matrigel and inoculated into 5-week-old male BALB/c nude mice (BALB/cAnN.Cg-*Foxn1^nu^*/CrlNarl) using intrahepatic injection with a dose of 1×10^5^ cells/50 μL per mouse. Tumor growth was monitored by bioluminescence imaging on an IVIS imaging system. Eight weeks later, mice were sacrificed to examine tumor growth in the liver. For subcutaneous inoculation, shGFP and shSCEL L2 cells (10^6^) were mixed with matrigel and injected 5-week-old male BALB/c nude mice (BALB/cAnN.Cg-*Foxn1^nu^*/CrlNarl). Left flank was injected with shGFP L2 cells, whereas right flank was injected with shSCEL L2 cells. Tumor size was measured by caliper weekly for seven weeks once tumors became visible. Tumor volume was calculated with the formula: (length x width^2^)/2.

### Statistical analysis

Data are expressed as means ± SD. The significance of difference was examined by Student's *t*-test (two-tailed). *P* < 0.05 was considered to be significant.

## SUPPLEMENTARY MATERIALS FIGURES AND TABLES


