# Supplementary material for: Sciellin mediates mesenchymal-to-epithelial transition in colorectal cancer hepatic metastasis
Source: Oncotarget. 2016 Mar 22;7(18):25742–54. doi: 10.18632/oncotarget.8264 (PMC5041940; doi:10.18632/oncotarget.8264)
Supplement: Supplementary file 1 [file oncotarget-07-25742-s001.pdf]

SUPPLEMENTARY MATERIALS

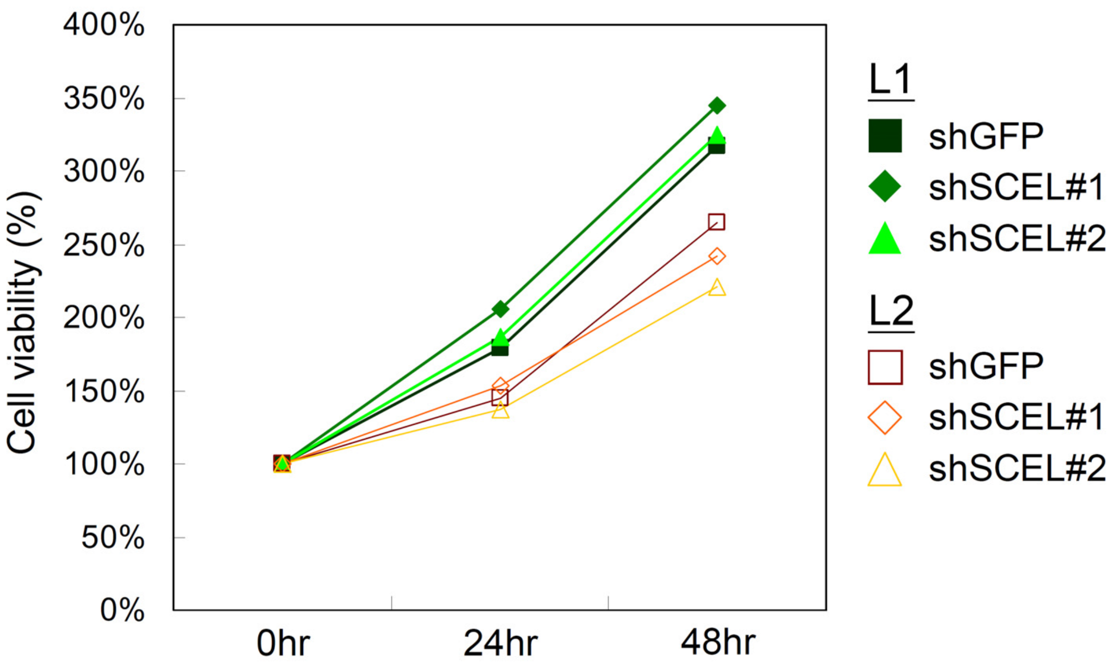

Supplementary Figure S1: Growth curve of L1 and L2 with shGFP (control) or shSCEL treatment.

Supplementary Table S1: The list of SCEL binding proteins

UniPort	Protein name	Gene name	MW [kDa]	Unique peptie
P62937	Peptidyl-prolyl cis-trans isomerase A	PPIA	18.01	5
P51571	Translocon-associated protein subunit delta	SSR4	20.21	4
Q14764	Major vault protein	MVP	99.33	4
Q99623	Prohibitin-2	PHB2	33.30	4
O75955	Flotillin-1	FLOT1	47.36	3
Q96S97	Myeloid-associated differentiation marker	MYADM	35.27	2
P83881	60S ribosomal protein L36a	RPL36A	17.73	3
Q9HCY8	Protein S100-A14	S100A14	11.66	4
O14818	Proteasome subunit alpha type-7	PSMA7	27.89	3
O60762	Dolichol-phosphate mannosyltransferase	DPM1	33.35	4
O00161	Synaptosomal-associated protein 23	SNAP23	23.35	2
O15145	Actin-related protein 2/3 complex subunit 3	ARPC3	20.55	2
Q06830	Peroxiredoxin-1	PRDX1	22.11	2
Q9UQB8	Brain-specific angiogenesis inhibitor 1-associated protein 2	BAIAP2	60.87	3
Q9H936	Mitochondrial glutamate carrier 1	SLC25A22	34.47	4
P49720	Proteasome subunit beta type-3	PSMB3	22.95	2
P60900	Proteasome subunit alpha type-6	PSMA6	28.15	3
Q8NC51	Plasminogen activator inhibitor 1 RNA-binding protein	SERBP1	43.14	4
P00367	Glutamate dehydrogenase 1, mitochondrial	GLUD1	61.40	2
P06733	Alpha-enolase	ENO1	47.17	2
Q9Y230	RuvB-like 2	RUVBL2	51.16	3
